# Interactions between the apolipoprotein E ε4 allele status and adverse childhood experiences on depressive symptoms in older adults

**DOI:** 10.3402/ejpt.v6.25178

**Published:** 2015-01-27

**Authors:** Subin Park, Yoon-Young Nam, Yoojin Sim, Jin Pyo Hong

**Affiliations:** 1 Department of Psychiatry, Seoul National Hospital, Seoul, South Korea; 2 Department of Psychiatry, Asan Medical Center, Ulsan University College of Medicine, Seoul, South Korea; 3 Department of Psychiatry, Samsung Medical Center, Seoul, South Korea

**Keywords:** Depression, old age, APOE-ε4, childhood adversity

## Abstract

**Background:**

The influence of childhood adversity on depression is modulated by genetic vulnerability. The apolipoprotein E ε4 (APOE-ε4) allele is a strong genetic risk factor for Alzheimer's disease (AD). Because late-life depressive symptoms could be a part of the preclinical course of AD, the APOE-ε4 allele may contribute to depression in old age.

**Objective:**

The aim of this study was to evaluate whether an APOE-ε4 carrier status was associated with depressive symptoms in older adults and to detect the gene–environment interaction between APOE-ε4 status and childhood adversity in relation to depressive symptoms in old age.

**Method:**

The participants consisted of 137 older adults (age range 50–70) without any psychiatric history or clinically significant cognitive impairment. APOE genotypes and measures of childhood adversity and depressive symptoms were obtained.

**Results:**

There was a significant positive association between adverse childhood experiences (ACE) scores and depressive symptoms (*B*=0.60; 95% CI=0.26, 0.93 for a 1 score increase in ACE scores; *p*=0.001). Although APOE-ε4 status per se was not associated with depressive symptoms, there was a significant interaction of the ACE scores with the APOE genotype in relation to depressive symptoms (*B*=0.78; 95% CI=0.02, 1.55; *p=*0.044). There was a significantly higher effect of childhood adversity on depressive symptoms in APOE-ε4 carriers than non-carriers (*t*=2.13, *p*=0.035).

**Conclusions:**

Our results suggest that the APOE-ε4 may modulate the association between childhood adversity and depressive symptoms in older adults. However, more research in a larger sample is needed to gain a better understanding of the relationship between the APOE-ε4, childhood adversity, and depression.

Depression is a complex phenotype that involves affective, motivational, cognitive, behavioral, and physical symptoms, as well as complex relationships between genetic and environmental factors (Levinson, 2006). Childhood adversity, including childhood abuse, neglect, and exposure to other traumatic stressors, has been described as one of the major environmental risk factors for adult depression (Chapman et al., 2004; Kendler, Kuhn, & Prescott, 2004). A large body of research has focused on identifying candidate genetic variations that interact with childhood adversity in predicting diagnoses, or symptom severity, of depression (Heim & Binder, 2012; Hornung & Heim, 2014; Klengel & Binder, 2013). A number of genes operating in neurobiological systems that mediate stress responses and/or synaptic plasticity, that is, the monoaminergic neurotransmitter, corticotropin-releasing hormone, glucocorticoid receptor-chaperone systems, neurotrophin, oxytocin, and endocannabinoid systems have been identified to date as significant moderators of the relationship between childhood adversity and depression (Hornung & Heim, 2014).

There is a strong association between the presence of apolipoprotein E ε4 (APOE-ε4) and increased risk of Alzheimer's disease (AD) (Kamboh, 1995). After the APOE-ε4 allele was established as a risk factor for AD, some investigators looked more closely at it as a possible risk factor for other neuropsychiatric disorders including late-onset depression (Martorell et al., 2001; Oliveri et al., 1999a, 1999b; Thibaut et al., 1998). While there are positive studies demonstrating associations between APOE-ε4 and depression in the elderly (Caselli et al., 2004; Flicker et al., 2004; Fritze et al., 2011; Krishnan et al., 1996; Rigaud et al., 2001; Stewart et al., 2001), there are also negative reports (Cantillon et al., 1997; Liu et al., 2002; Mauricio et al., 2000; Schmand, Hooijer, Jonker, Lindeboom, & Havekes, 1998; Surtees et al., 2009).

It has been suggested that the APOE-ε4 may influence response to both physiologic and psychological stressors (Roses et al., 1996). Animal studies have suggested that there is an altered response to physiologic stressors and neurotoxic stimuli in mice with deletion of the murine APOE gene (Gordon, Ben-Eliyahu, Rosenne, Sehayek, & Michaelson, 1996) or with transgenic expression of the human APOE-ε4 isoform (Buttini et al., 2000). Human studies have also indicated that APOE-ε4 carriers were more vulnerable to adverse cognitive consequences of stress than non-carriers (Comijs, Van den Kommer, Minnaar, Penninx, & Deeg, 2011; Lee et al., 2008, 2011; Petkus, Wetherell, Stein, Liu, & Barrett-Connor, 2012).

Although several studies suggested the interactive effect of stressor and APOE genotype on cognitive function (Comijs et al., 2011; Lee et al., 2008, 2011; Petkus et al., 2012), we are aware of only one study that has investigated the interactive effect of psychosocial stress and APOE genotype on depression or depressive symptoms. In a study in healthy adult women, Gallagher-Thompson, O'Hara, Simmons, Kraemer, and Murphy (2001) found increased levels of self-reported stress to be associated with increased levels of depression in APOE-ε4 carriers, but not in non-carriers, suggesting that APOE-ε4 carriers have stronger responses to stress than non-carriers. From the perspective of gene–environment interaction, the APOE-ε4 allele may increase vulnerability of the aging brain to adverse effects of life stress including childhood adversity.

On the basis of this previous research, the aim of the present study was to evaluate whether APOE-ε4 carrier status is associated with depressive symptoms in older adults and to detect the gene–environment interaction between the APOE-ε4 status and childhood adversity in relation to depressive symptoms in old age.

## Methods

### Participants

The present study consisted of 137 adults (82 women) aged 50–70 without dementia or severe cognitive impairment. Participants were recruited from a community health center in Seoul. All participants were enrolled in the study by responding to an advertisement. Patients with dementia or severe cognitive impairment were excluded through clinical interview by clinicians. All participants were ethnically Korean. After the study details had been fully explained, written informed consent to participate was obtained from each participant. Participants completed several self-report questionnaires in the presence of the study coordinator. The study protocol was approved by the Institutional Review Board or Ethics Committee of Asan Medical Center.

### Measures

To measure childhood adversity, we used the adverse childhood experiences (ACE) questionnaire developed by the ACE study group of the Centers for Disease Control and Prevention (Dong, Anda, Dube, Giles, & Felitti, 2003; Centers for Disease Control and Prevention, 1998), which included detailed information about ACE and family and household dysfunction. The questionnaire consists of five categories of childhood abuse (emotional abuse, physical abuse, sexual abuse, emotional neglect, and physical neglect) and five categories of exposure to household dysfunction during childhood (battered mother, household substance abuse, mental illness in household, parental separation or divorce, and criminal household member). All ACE questions referred to the respondents’ first 18 years of life. Respondents were defined as exposed to a category if they responded “yes” to one or more of the questions in that category. The ACE score was the sum of categories with an exposure; thus, the possible number of exposures ranged from 0 (unexposed) to 10 (exposed to all categories). A previous study has found a high internal consistency (Cronbach's alpha=0.88 for the 10 discrete binary items) (Murphy et al., 2014). In addition, when a respondent was exposed to one of the ACE, the probability of exposure to any other category of ACE increased substantially (Dube et al., 2003; Murphy et al., 2014). The ACE were translated (including back-translation) into Korean and used in this study.

To measure the severity of depressive symptoms, we used the Korean Self-Rating version of the Quick Inventory of Depressive Symptomatology (QIDS-SR) (Hong, Parker, Park, Lim, & Jeon, 2013). This questionnaire contains 16 items measuring nine criterion symptom domains (sleep, sad mood, appetite/weight, concentration/decision making, self-view, thoughts of death or suicide, general interest, energy level, and restlessness/agitation) that define a major depressive episode according to the Diagnostic Statistical Manual for Mental Disorders-4th edition (DSM-IV). The scores for three domains (sleep, appetite/weight, and restlessness/agitation) are based upon the maximum score (most pathological) of two or more questions. Each of the remaining domains is rated by a single item. All domains are scored from 0 to 3, with higher scores reflecting greater psychopathology. Total QIDS-SR scores range from 0 to 27. In a study of 596 adult outpatients with chronic, non-psychotic major depressive disorder, Rush et al. (2003) found high correlations among the QIDS-SR and the Hamilton Rating Scale for Depression (HRSD) 17-item version (*c*=0.81), HRSD 21-item version (*c*=0.82), and HRSD 24-item version (*c*=0.84), and a high internal consistency (Cronbach's alpha=0.86). In a validation study of the Korean QIDS-SR (Hong et al., 2013), the Cronbach's alpha coefficient was 0.73 and correlations of the QIDS-SR with the HRSD, Patient Health Questionnaire-9, and the Center for Epidemiologic Studies were 0.78, 0.81, and 0.76, respectively. There were no missing values on individual items of ACE and QIDS-SR.

### APOE genotype

Genomic DNA was prepared from peripheral blood samples using a nucleic acid isolation device, QuickGene-mini80 (FUJIFILM, Tokyo, Japan). The SNaPshot assay was performed according to the manufacturer's instructions (ABI PRISM SNaPShot Multiplex kit, Foster City, CA, USA). Analysis was carried out using GeneMapper software (version 4.0; Applied Biosystems). Primer sets and Tm used for the SNaPshot are as follows: forward primer GCGGACATGGAGGACGTG, reverse primer CTGGGCCCGCTCCTGTAG, and genotyping primer CGCGATGCCGATGACCTGCAGAAG for APOE(rs7412) and forward primer GCGGACATGGAGGACGTG, reverse primer CTGGGCCCGCTCCTGTAG, and genotyping primer GCGGACATGGAGGACGTG for APOE(rs429358).

### Statistical analysis

Demographic and clinical characteristics were compared between APOE-ε4 carriers and non-carriers using an independent *t*-test for continuous variables and a chi-square test for categorical variables. We explored the modulation of the relationship between ACE scores and depressive symptoms in old age by APOE-ε4 carrier status using Baron and Kenny's method. A multiple linear regression model was constructed with QIDS-SR scores as the dependent variable and the APOE-ε4 carrier status, ACE scores, sex, age, years of education, and marital and occupational status as independent variables. Subsequently, the modulation of APOE-ε4 carrier status on the relationship between childhood trauma and depressive symptoms in old age was explored by including an interaction term in the models. An interaction term was constructed by multiplying the two main components, ACE scores (continuous variable) and APOE-ε4 carrier status (0=absence of ε4 allele, 1=presence of ε4 allele). To meet the assumptions of the model, all variables except age, educational years, and ACE scores (continuous variables) were converted into dummy variables. Difference in ACE slopes between genotypes was tested for the interaction term between ACE scores and APOE-ε4 carrier status using a multiple linear regression analysis. SPSS (version 21.0; SPSS Inc., Chicago, IL) was used to perform all statistical analyses and a *p*-value less than 0.05 was considered significant.

## Results

Among our 137 study participants, 21 carried the APOE-ε4 allele. Sixty-one participants (44.5%) had depressive symptoms based on QIDS-SR: 48 had mild symptoms (QIDS-SR scores, 6–10) and 13 had moderate symptoms (QIDS-SR scores, 11 or above). There were no significant differences in demographic characteristics, ACE scores, and QIDS-SR scores between APOE-ε4 carriers and non-carriers (Table 1). Table 2 shows the modulation of the relationship between ACE and depressive symptoms in older persons by APOE-ε4 carrier status. Model 1, which included APOE-ε4 carrier status, ACE scores, sex, age, educational years, and marital and occupational status as independent variables, revealed a significant positive association between ACE scores and depressive symptoms in older adults (unstandardized regression coefficient, *B=*0.54; 95% CI=0.20, 0.88 for a 1 score increase in ACE scores; *p*=0.002) but no significant association between ACE scores and APOE genotype (*B=*1.55; 95% CI=−0.54, 3.64; *p*=0.145). Model 2, which included an interaction term to investigate the possible modulation of ACE by APOE-ε4 carrier status, revealed a significant interaction of the ACE scores with the APOE genotype in relation to QIDS-SR scores (*B*=0.85; 95% CI=0.10, 1.61; *p=*0.027). For subjects with the APOE-ε4 allele, there was a positive and significant association of the ACE scores with the QIDS-SR scores (*B=*1.17; 95% CI=0.52, 1.82 for a 1 score increase in ACE scores, *p*=0.001). However, for those subjects without the APOE-ε4 allele, the association between ACE scores and QIDS-SR scores was positive but not statistically significant (*B=*0.32; 95% CI=−0.07, 0.71 for a 1 score increase in ACE scores, *p*=0.107).

**Table 1:** Characteristics of the study participants according to their apolipoprotein ε-4 (APOE-ε4) carrier status

	Non-carriers (*N=*116)	Carriers (*N=*21)	Total	Test statistics^a^	Effect size^a^	*p* a
Gender, female, *n* (%)	69 (59.5)	13 (61.9)	82 (59.9)	0.04^b^	0.02^c^	0.835
Age (years), mean (SD)	59.01 (6.00)	60.38 (6.73)	59.22 (6.11)	−0.95^d^	−0.21^e^	0.346
Educational years, mean (SD)	12.30 (4.10)	11.33 (4.04)	12.15 (4.10)	0.99^c^	0.24^e^	0.323
Marital status				0.46^b^	0.06^d^	0.550
Married	92 (79.3)	18 (85.7)	110 (80.3)			
Widowed/divorced/separated	24 (20.7)	3 (14.3)	27 (19.7)			
Employment				0.36^b^	0.05^d^	0.547
Employed	58 (50.0)	9 (42.9)	67 (48.9)			
Unemployed	58 (50.0)	12 (57.1)	70 (51.1)			
Apolipoprotein genotype, *n*				NA	NA	NA
ε2/ε3	16	0	16			
ε3/ε3	100	0	100			
ε3/ε4	0	21	21			
ACE score (0–10)	1.16 (2.23)	1.86 (2.99)	1.26 (2.36)	0.99^c^	−0.04^e^	0.323
QIDS-SR score (0–27)	7.17 (5.07)	8.71 (5.43)	7.41 (5.15)	−1.27^c^	−0.29^e^	0.207

NA=not applicable; ACE=adverse childhood experiences; QIDS-SR=Quick Inventory of Depressive Symptomatology-Self Rating.

a Comparison between non-carriers and carriers.

b Chi-square test analysis.

c Φ analysis.

d *t*-statistics analysis.

e Cohen's d analysis.

**Table 2:** Results of multiple linear regression models exploring the effect of childhood adversity and an apolipoprotein-ε4 (APOE-ε4) carrier status on depressive symptoms

	*B* (95% CI)	SE	*t*	*p*
Model 1: Overall				
Intercept	−0.04 (−11.01, 10.92)	5.54	−0.01	0.994
ACE scores	0.54 (0.20, 0.88)	0.17	3.14	0.002
APOE-ε4 carrier status (for ACE=0)	1.55 (−0.54, 3.64)	1.06	1.46	0.145
Model 2				
Non-carrier				
Intercept	1.37 (−9.50, 12.23)	5.49	0.25	0.804
ACE scores	0.32 (−0.07, 0.71)	0.20	1.63	0.107
Carrier				
Intercept	1.56 (−9.42, 12.54)	5.55	0.28	0.779
ACE scores	1.17 (0.52, 1.82)	0.33	3.56	0.001
APOE-ε4 carrier status (for ACE=0)	0.19 (−2.19, 2.58)	1.21	0.16	0.874
ACE×APOE-ε4	0.85 (0.10, 1.61)	0.38	2.24	0.027

*B*=unstandardized regression coefficient; CI=confidence interval; SE=standard error; ACE=adverse childhood experiences. Independent variables are displayed in the first column and the dependent variables are the Quick Inventory of Depressive Symptomatology-Self Rating (QIDS-SR) scores. Model 1 includes sex, age, educational years, marital and occupational status, ACE scores, and APOE-ε4 carrier status as independent variables; Model 2 includes an ACE×APOE-ε4 interaction term in addition to the variables in model 1. The intercept is the value when all the adjustment variables have a value of 0 (i.e., sex=male, age=0 year, educational years=0, marital status=married, occupational status=employed). *F*=7.50, *p<*0.001, and *R*^2^=0.28 in Model 1, and *F*=7.43, *p<*0.001, and *R*^2^=0.30 in Model 2. The proportions of floor and ceiling values were 58.4 and 0% for ACE and 3.6 and 0% for QIDS-SR.

## Discussion

Our present results show that childhood adversity may be a predictor of adult depressive symptoms, in accordance with previous clinical and epidemiological reports (Chapman et al., 2004; Kendler et al., 2004). From the perspective of gene–environment interactions, we found that childhood adversity was associated with increased levels of depression only in individuals with the APOE-ε4 allele, suggesting that childhood adversity may more strongly impact depressive symptomatology in old age in APOE-ε4 carriers than in APOE-ε4 non-carriers.

The proportion of subjects with APOE-ε4 allele (15%) was similar to that in previous studies of general populations (Kim et al., 2010 [15.6%]; Kim et al., 2004 [17.5%]) or depressed populations (Kim et al., 2011 [18.4%]). Previous researches suggested that the APOE-ε4 allele frequency in the depressed group was not significantly different from that in the controls (Kim et al., 2010; Rigaud et al., 2001).

Several authors undertook studies of the APOE genotype in late-life depression, with mixed results. Some researchers found that the APOE-ε4 allele frequency of older depressed patients was elevated relative to normal controls (Krishnan et al., 1996; Rigaud et al., 2001; Stewart et al., 2001). The ε4 allele was reported to be associated with increased depressive symptoms in community-dwelling older men (Flicker et al., 2004). However, other researchers found no association between APOE genotype and depression (Schmand et al., 1998; Surtees et al., 2009) or depressive symptoms (Liu et al., 2002; Locke et al., 2013; Mauricio et al., 2000) in community-dwelling older adults. Our current results also did not indicate that APOE-ε4 carrier status per se is associated with depressive symptoms in old age.

Consistent with a previous study that found increased levels of stress to be associated with increased levels of depression only in APOE-ε4 carriers (Gallagher-Thompson et al., 2001), we found that the risk of depressive symptoms related to childhood adversity increased with the presence of the APOE-ε4 allele. Previous studies reported that APOE-ε4 was associated with low plasma amyloid-β peptide 42 (Aβ42) in older adults (Qiu et al., 2007; Sun et al., 2009). Plasma Aβ42 declines significantly in the preclinical stage of AD (Irizarry, 2004; Mayeux et al., 2003; Pomara, Willoughby, Sidtis, & Mehta, 2005; Solfrizzi et al., 2006), suggesting that reduced plasma Aβ42 plays a role in AD pathogenesis or is a biomarker predicting the onset of AD. Although the meaning of the relationship between depression and a low concentration of Aβ42 in plasma is unclear, low plasma Aβ42 is reported to be associated with a depression subtype in old age, namely amyloid-associated depression (Qiu et al., 2007). It can be suggested that APOE-ε4 carrier status per se is not predictive for late-life depressive symptoms but engenders vulnerability to reactive depression following childhood adversity. Although the mechanism by which APOE-ε4 increases such vulnerability is unclear, lower plasma Aβ42 in APOE-ε4 carriers compared to non-carriers may have a role.

There are some limitations to our present study that should be mentioned. First, the cross-sectional nature of our study design did not allow causal associations to be robustly tested. Although some studies have found high reliability of self-reports of childhood trauma (e.g., Fink et al., 1995), the retrospective measure of childhood adversity may be influenced by recall bias. Second, we did not use a reliable dementia screening measure to exclude dementia or severe cognitive impairment. Third, the sample size of the present study was relatively small for genotypic analysis and the results should therefore be carefully interpreted. Furthermore, the sample was recruited by responses to an advertisement, and is therefore not likely to be representative for any well-defined population. Fourth, there might be behavioral and health factors (e.g., tobacco and alcohol use, cardiovascular disease), not accounted for in this study, which co-vary with childhood adversity and could impact risk for the development of depressive symptoms. Finally, the population was not a clinically depressed one; thus, further work is needed in a clinical population with significant levels of depression to confirm our findings.

Despite these limitations, our current findings demonstrate for the first time that there may be a modulating effect of APOE-ε4 on the association between ACE and late-life depressive symptoms. Further studies of a larger sample size with a prospective design are warranted to further evaluate the APOE-ε4×ACE interaction effect on late-life depression, although prospective studies of consequences of childhood adversities in old age will take quite some time.

## Supplementary Material

Interactions between the apolipoprotein E ε4 allele status and adverse childhood experiences on depressive symptoms in older adults

Interactions between the apolipoprotein E ε4 allele status and adverse childhood experiences on depressive symptoms in older adults

Interactions between the apolipoprotein E ε4 allele status and adverse childhood experiences on depressive symptoms in older adults

Interactions between the apolipoprotein E ε4 allele status and adverse childhood experiences on depressive symptoms in older adults
