# Supplementary material for: Interactions between the apolipoprotein E ε4 allele status and adverse childhood experiences on depressive symptoms in older adults
Source: Eur J Psychotraumatol. 2015 Jan 27;6:10.3402/ejpt.v6.25178. doi: 10.3402/ejpt.v6.25178 (PMC4309830; doi:10.3402/ejpt.v6.25178)
Supplement: Interactions between the apolipoprotein E ε4 allele status and adverse childhood experiences on depressive symptoms in older adults [file EJPT-6-25178-s001.pdf]

Interaktion zwischen Apoliopoprotein E ε4 und aversiven Kindheitserlebnissen im Hinblick auf depressive Symptomatik im fortgeschrittenen Erwachsenenalter

Subin Park, Jin Pyo Hong

Hintergrund: Der Einfluss aversiver Kindheitserlebnisse auf Depressionen durch die genetische Vulnerabilität moderiert. Das Apoliopoprotein E ε4 (APOE-ε4) Allel ist ein starker Risikofaktor für die Alzheimer-Erkrankung. Da depressive Symptomatik in späteren Lebensjahren Teil einer vorklinischen Alzheimer-Erkrankung sein könnte, besteht die Möglichkeit, dass das APOE-ε4 Allel zu einer depressiven Symptomatik beiträgt.
Ziel: Ziel der Studie ist es, die Verbindung von depressiven Symptomen im späteren Erwachsenenalter und dem APOE-ε4 Träger-Status zu untersuchen, sowie die Anlage- Umwelt-Interaktion zwischen Träger-Status und Erfahrungen in der Kindheit im Hinblick auf spätere Depressionen zu erforschen.
Methode: Die 137 TeilnehmerInnen (50-70 Jahre alt) wiesen keine psychiatrische Vorgeschichte oder signifikante kognitive Einschränkung auf. Der APOE-ε4 Träger-Status wurde erhoben, ebenso aversive Erfahrungen in der Kindheit und depressive Symptome.
Ergebnisse: Es wurde eine signifikante Korrelation zwischen aversiven Erfahrungen in der Kindheit und depressiven Symptomen im späten Erwachsenenalter gefunden ($B = 0.60$; 95% CI = 0.26, 0.93 für 1 Punktanstieg bei aversiven Erfahrungen in der Kindheit; $p = 0.001$). Obwohl der APOE-ε4 Träger-Status an sich nicht mit depressiven Symptomen korreliert war, gab es eine signifikante Interaktion zwischen aversiven Kindheitserfahrungen und der Genetik im Hinblick auf spätere depressive Symptome ($B = 0.78$; 95% CI = 0.02, 1.55; $p = 0.044$). Der Effekt von aversiven Kindheitserlebnissen auf spätere depressive Symptomatik war signifikant höher bei APOE-ε4 Trägern im Vergleich zu Nicht-Trägern.
Schlussfolgerung: Unsere Ergebnisse lassen den Schluss zu, dass das APOE-ε4 Allel die Verbindung von aversiven Kindheitserloebnissen und späteren depressiven Symptomen moduliert. Mehr Forschung und eine größere Stichprobe erscheinen notwendig um die Verbindung von APOE-ε4, Depressionen und Kindheitserlebnissen zu verstehen.

Keywords/Schlagwörter: Depressionen, höheres Alter, APOE-ε4, aversive Kindheitserlebnisse
Name of translator: Iris Fischer, Brigitte Lueger-Schuster

Citation: European Journal of Psychotraumatology 2015, 6: 25178 - http://dx.doi.org/10.3402/ejpt.v6.25178
