# Supplementary material for: Interactions between the apolipoprotein E ε4 allele status and adverse childhood experiences on depressive symptoms in older adults
Source: Eur J Psychotraumatol. 2015 Jan 27;6:10.3402/ejpt.v6.25178. doi: 10.3402/ejpt.v6.25178 (PMC4309830; doi:10.3402/ejpt.v6.25178)
Supplement: Interactions between the apolipoprotein E ε4 allele status and adverse childhood experiences on depressive symptoms in older adults [file EJPT-6-25178-s002.pdf]

Yaşlı yetişkinlerin depresif belirtilerinde Apolipoprotein E ε4 allel durumu ve olumsuz çocukluk çağı deneyimleri etkileşimi

Subin Park, Jin Pyo Hong

Arkaplan: Çocukluk çağı zorluklarının depresyon üzerindeki etkisi genetik yatkınlığa bağlı olarak değişmektedir. Apolipoprotein E ε4 (APOE-ε4) alleli Alzheimer hastalığı için güçlü bir genetik risk faktörüdür. İleri yaşam dönemlerindeki depresif belirtiler Alzheimer hastalığının preklinik döneminin bir parçası olabileceği için, APOE-ε4 allelinin ileri yaş depresyonunda payı olabilir.
Amaç: Çalışmanın amacı, APOE-ε4 taşıyıcılık durumunun yaşlı bireylerde depresif belirtiler ile ilgisi olup olmadığını araştırmak ve ileri yaştaki depresif belirtilerle ilgili olarak APOE-ε4 durumu ve çocukluk çağı zorlukları arasındaki gen-çevre etkileşimini saptamaktır.
Yöntem: Katılımcılar, psikiyatrik hikayesi veya klinik olarak anlamlı bir bilişsel bozulması olmayan 137 yaşlı yetişkinden (yaş aralığı 50-70) oluşmaktadır. APOE genotipleri, çocukluk çağı zorlukları ve depresif belirti ölçüleri elde edilmiştir.
Sonuçlar: Olumsuz çocukluk çağı deneyimleri (ACE) ve depresif belirtiler arasında anlamlı ve olumlu bir ilişki vardır ($B = 0.60$; 95% CI = 0.26, 0.93 ACE skorlarındaki 1 puan artma için; $p = 0.001$). APOE-ε4 durumu kendi başına depresif belirtilerle ilişkili olmasa da, depresif belirtilerle ilgili olarak ACE skorları ve APOE genotipi arasında anlamlı bir etkileşim vardır ($B = 0.78$; 95% CI = 0.02, 1.55; $p = 0.044$). APOE-ε4 taşıyıcılarında, taşıyıcı olmayanlara göre çocukluk çağı zorluklarının depresif belirtiler üzerindeki etkisi daha büyüktür ($t = 2.13$, $p = 0.035$).
Tartışma: Sonuçlarımız, yaşlı yetişkinlerde APOE- ε4'nin çocukluk çağı zorlukları ve depresif belirtiler arasındaki ilişkiyi düzenlediğini ortaya koymuştur. Ancak, APOE- ε4, çocukluk çağı zorlukları ve depresyon arasındaki ilişkiyi daha iyi anlayabilmek için daha büyük bir örneklemde daha fazla araştırma yapılması gerekmektedir.

Anahtar kelimeler: depresyon; ileri yaş; APOE- ε4; çocukluk çağı zorlukları

Name of translator: Emek Yuce Zeyrek-Rios

Citation: European Journal of Psychotraumatology 2015, 6: 25178 - http://dx.doi.org/10.3402/ejpt.v6.25178
