# Supplementary material for: Interactions between the apolipoprotein E ε4 allele status and adverse childhood experiences on depressive symptoms in older adults
Source: Eur J Psychotraumatol. 2015 Jan 27;6:10.3402/ejpt.v6.25178. doi: 10.3402/ejpt.v6.25178 (PMC4309830; doi:10.3402/ejpt.v6.25178)
Supplement: Interactions between the apolipoprotein E ε4 allele status and adverse childhood experiences on depressive symptoms in older adults [file EJPT-6-25178-s003.pdf]

Взаимосвязь депрессивных симптомов в пожилом возрасте с состоянием аллеля ε4 аполипопротеина Е и негативным детским опытом

Subin Park, Jin Pyo Hong

История вопроса: Влияние неблагоприятной обстановки в детстве на депрессию модулируется генетической уязвимостью. Аллель ε4 аполипопротеина Е (АПОЕ-ε4) – сильный генетический фактор риска для болезни Альцгеймера. Поскольку на поздних этапах жизни депрессивные симптомы могут быть частью доклинических стадий заболевания Альцгеймера, АПОЕ-ε4 может способствовать депрессии в пожилом возрасте.
Цель: В рамках данного исследования планировалось оценить, насколько носитель аллеля АПОЕ-ε4 был связан с депрессивными симптомами у пожилых людей, а также обнаружить на генном уровне взаимосвязь депрессивных симптомов в пожилом возрасте с состоянием АПОЕ-ε4 и неблагоприятной обстановкой в детстве.
Метод: В исследовании приняли участие 137 пожилых людей (возрастной диапазон 50-70 лет) без какой-либо психиатрической истории или клинически значимых когнитивных нарушений. Были получены генотипы АПОЕ, измерены депрессивные симптомы и оценен уровень неблагоприятной обстановки в детстве.
Результаты: Была получена значимая положительная связь между неблагоприятным детским опытом (НДО) и депрессивными симптомами (B = 0,60; 95% CI = 0,26, 0,93 на 1 балл прироста по шкале НДО; p = 0,001). Хотя статус АПОЕ-ε4 как таковой не был связан с депрессивными симптомами, была получена значимая взаимосвязь шкалы НДО с генотипом АПОЕ по отношению к депрессивным симптомам (B = 0,78; 95% CI = 0,02, 1,55; p = 0,044). Эффект неблагоприятной обстановки в детстве на депрессивные симптомы был значительно выше у носителей АПОЕ-ε4, чем у не носителей (t = 2,13, p = 0,035).
Выводы: Наши результаты показывают, что АПОЕ-ε4 может модулировать связь между неблагоприятной обстановкой в детстве и депрессивными симптомами у пожилых людей. Тем не менее, необходимы дополнительные исследования в большей выборке, что нужно для лучшего понимания взаимосвязи между АПОЕ-ε4, неблагоприятной обстановкой в детстве и депрессией.

Ключевые слова: депрессия, старость, АПОЕ-ε4, неблагоприятная обстановка в детстве.

Name of translator: Marina Scherbak

Citation: European Journal of Psychotraumatology 2015, 6: 25178 - http://dx.doi.org/10.3402/ejpt.v6.25178
