# Supplementary material for: Interactions between the apolipoprotein E ε4 allele status and adverse childhood experiences on depressive symptoms in older adults
Source: Eur J Psychotraumatol. 2015 Jan 27;6:10.3402/ejpt.v6.25178. doi: 10.3402/ejpt.v6.25178 (PMC4309830; doi:10.3402/ejpt.v6.25178)
Supplement: Interactions between the apolipoprotein E ε4 allele status and adverse childhood experiences on depressive symptoms in older adults [file EJPT-6-25178-s004.pdf]

Interactions between the apolipoprotein E ε4 allele status and adverse childhood experiences on depressive symptoms in older adults

Subin Park, Jin Pyo Hong

Interakcja pomiędzy allejami genu ES4 i negatywnymi doświadczeniami z dzieciństwa a poziomem objawów depresji wsród osób dorosłych w starszym wieku. Wprowadzenie: Wielu autorów wykazało, że związek między negatywnymi doświadczeniami w dzieciństwie i depresją jest moderowany przez podatność genetyczną. Alleje genu ES4 (APOE- S4) są silnymi genetycznymi czynnikami ryzyka choroby Alzheimera. Ponieważ symptomy depresyjne w późnym wieku mogą poprzedzać chorobe Alzheimera, APOE-S4 może przyczynić się do wystepowania depresji w późnym wieku.
Cel: Celem niniejszej pracy było sprawdzenie, czy istnieje związek pomiędzy APOE-S4 a występowaniem symptomów depresyjnych wśród dorosłych w starszym wieku oraz określenie genetyczno-środowiskowej interakcji pomiędzy APOE-S4 a negatywnymi doświadczeniami w dzieciństwie.
Metoda: Zbadano 137 dorosłych w starszym wieku (50-70 lat) bez żadnej historii zaburzeń psychicznych lub klinicznie zdiagnozowanego uposledzenia funkcji poznawczych. Zmierzono poziom symptomów depresyjnych, negatywne doświadczenia z dzieciństwa oraz opisane wyżej czynniki genetyczne.
Wyniki: Zaobserwowano istotnie statystyczne związek pomiędzy negatywnymi doświadczeniami z dzieciństwa oraz poziomem symptomów depresyjnych. Choć APOE-S4 jako taki nie był bezpośrednio zwiazany z poziomem objawów depresji, to jednak odnotowano istotną interakcję pomiedzy APOE-S4 i negatywnymi doświadczeniami z dzieciństwa a nasileniem depresji wśród badanych. Konkluzje: Wyniki naszych badań wskaują, że APOE-S4 może moderować związek pomiędzy negatywnymi doświadczeniami z dzieciństwa a nasileniem depresji wśród osób badanych. Jednakże istnieje konieczność przeprowadzeie badań na większych próbach, by uzyskać bardziej rzetelne dane na temat powyzszej tematyki.

Keywords: depresja; starszy wiek; APOE-ε4; negatywne doświadczenia z dzieciństwa.

Name of translator: Marcin Rzeszutek, University of Finance and Management in Warsaw, Poland

Citation: European Journal of Psychotraumatology 2015, 6: 25178 - http://dx.doi.org/10.3402/ejpt.v6.25178
